# 局限期小细胞肺癌放疗靶区前瞻性随机对照研究的初步报告

**DOI:** 10.3779/j.issn.1009-3419.2010.07.07

**Published:** 2010-07-20

**Authors:** 晓 胡, 勇 包, 力 张, 媛媛 陈, 凯新 李, 卫华 王, 源 刘, 瀚 何, 宗文 孙, 婷婷 庄, 彦 王, 静 陈, 颖 梁, 阳 张, 洪云 赵, 凤华 王, 明 陈

**Affiliations:** 1 510060 广州，华南肿瘤学国家重点实验室，中山大学肿瘤防治中心放疗科 State Key Laboratory of Oncology in Southern China, Department of Radiation Oncology, Sun Yat-Sen University Cancer Center, Guangzhou 510060, China; 2 510060 广州，中山大学肿瘤防治中心内科 Department of Medical Oncology, Sun Yat-Sen University Cancer Center, Guangzhou 510060, China

**Keywords:** 肺肿瘤, 小细胞肺癌, 局限期, 放疗靶区, Lung neoplasms, Small cell lung cancer, Limited-stage, Radiation target volume

## Abstract

**背景与目的:**

在局限期小细胞肺癌（limited-stage small cell lung cancer, LSCLC）的放化疗综合治疗中，放疗靶区等方面尚存争议。本研究旨在前瞻性比较LSCLC经诱导化疗后按不同靶区范围进行放疗的局部控制率和毒副反应的差异及对生存的影响。

**方法:**

LSCLC患者，经EP方案诱导化疗2周期后，随机分为研究组和对照组，分别按照化疗后和化疗前原发灶范围勾画放疗靶区（gross tumor volume-tumor, GTV-T），区域淋巴结靶区（gross tumor volume-nodal, CTV-N）两组均包括达到诊断标准的淋巴结所在的结区。放疗45 Gy/30次/19天，开始于化疗后1周-2周，放疗中按期进行第3周期化疗。放疗后再行3周期化疗。完全缓解者行预防性全脑照射（prophylactic cranial irradiation, PCI）。

**结果:**

研究组与对照组分别入组37例、40例患者。局部复发率分别为32.4%、28.2%（*P*=0.80），其中单独照射野外复发率分别为3.0%、2.6%（*P*=0.91），且均位于原发病灶同侧锁骨上区。纵隔型N3是照射野外复发危险因素（*P*=0.02, OR=14.13, 95%CI: 1.47-136.13）；放疗期间发生Ⅰ度、Ⅱ度体重减轻分别为29.4%、5.9%和56.4%、7.7%（*P*=0.04）；0度-Ⅰ度和Ⅱ度-Ⅲ度后期放射性肺损伤发生率分别为97.1%、2.9%和84.6%、15.4%（*P*=0.07）。研究组和对照组中位生存时间分别为22.1个月和26.9个月；1、2、3年总生存率分别为77.9%、44.4%、37.3%及75.8%、56.3%、41.7%（*P*=0.79）。

**结论:**

本研究结果显示仅照射化疗后原发灶范围及阳性淋巴结区未降低局部控制率和总生存率，而放疗毒性降低。但目前样本量尚未达到设计要求，最终结论需继续扩大样本数后得出。

## Abstract (en)

**Background and objective:**

Controversies exists with regard to target volumes as far as thoracic radiotherapy (TRT) is concerned in the multimodality treatment for limited-stage small cell lung cancer (LSCLC). The aim of this study is to prospectively compare the local control rate, toxicity profiles, and overall survival (OS) between patients received different target volumes irradiation after induction chemotherapy.

**Methods:**

LSCLC patients received 2 cycles of etoposide and cisplatin (EP) induction chemotherapy and were randomly assigned to receive TRT to either the postor pre-chemotherapy tumor extent (GTV-T) as study arm and control arm, CTV-N included the positive nodal drainage area for both arms. One to 2 weeks after induction chemotherapy, 45 Gy/30 Fx/19 d TRT was administered concurrently with the third cycle of EP regimen. After that, additional 3 cycles of EP consolidation were administered. Prophylactic cranial irradiation (PCI) was administered to patients with a complete response.

**Results:**

Thirty-seven and 40 patients were randomly assigned to study arm and control arm. The local recurrence rates were 32.4% and 28.2% respectively (*P*=0.80); the isolated nodal failure (INF) rates were 3.0% and 2.6% respectively (*P*=0.91); all INF sites were in the ipsilateral supraclavicular fossa. Medastinal N3 disease was the risk factor for INF (*P*=0.02, OR=14.13, 95%CI: 1.47-136.13). During radiotherapy, grade Ⅰ, Ⅱ weight loss was observed in 29.4%, 5.9% and 56.4%, 7.7% patients respectively (*P*=0.04). Grade 0-Ⅰ and Ⅱ-Ⅲ late pulmonary injury was developed in 97.1%, 2.9% and 86.4%, 15.4% patients respectively (*P*=0.07). Median survival time was 22.1 months and 26.9 months respectively. The 1 to 3-year OS were 77.9%, 44.4%, 37.3% and 75.8%, 56.3%, 41.7% respectively (*P*=0.79).

**Conclusion:**

The preliminary results of this study indicate that irradiant the post-chemotherapy tumor extent (GTV-T) and positive nodal drainage area did not decrease local control and overall survival while radiation toxicity was reduced. But the current sample size has not met designed requirements, and further investigation is warranted before final conclusions could be drawn.

肺癌是常见胸部恶性肿瘤，临床所见肺癌患者中约15%-20%为小细胞肺癌（small cell lung cancer, SCLC），其中40%为局限期（limited-stage small cell lung cancer, LSCLC）患者。SCLC对放化疗均敏感，然而，单独接受化疗的LSCLC患者局部复发率高达75%-90%^[1]^。胸部放射治疗的引入，使局部复发率降低至50%，且使LSCLC患者的长期生存率提高5%^[2, 3]^。因此放疗与化疗综合治疗成为LSCLC的标准治疗方案。

目前，LSCLC化疗仍以EP方案为标准方案^[4]^。尽管Turrisi等^[5]^的Ⅲ期临床试验采用的45 Gy/30次/19天加速超分割放疗第1天起联合EP方案同步化疗取得了目前为止最佳的疗效：5年生存率为26%，中位生存时间为26个月，但在临床实践中，我们仍然会面对多程诱导化疗后的患者。此外，国际上大多数关于LSCLC放化综合治疗的前瞻性临床试验设计也采用诱导化疗联合放疗^[6-13]^。而二十多年来，诱导化疗后的LSCLC胸部放疗范围一直存在争议，鲜有关于这方面的前瞻性临床研究^[14]^。

中山大学肿瘤防治中心胸部放疗组自2002年6月起针对LSCLC放疗靶区开展前瞻性随机对照临床研究。将放疗靶区随机分为按诱导化疗后或诱导化疗前原发灶范围勾画，两组的纵隔淋巴引流区的靶区只包括达到诊断标准的阳性淋巴结所在的结区，不进行预防性照射。

## 1 资料与方法

### 1.1 病例选择

#### 1.1.1 入组标准

经病理或细胞学确诊为SCLC；经影像学证实为LSCLC（包括脑增强MRI或CT、胸腹部增强CT、全身骨扫描），LSCLC按美国退伍军人医院定义^[15]^，并进一步行TNM分期；年龄≥18岁且≤75岁，既往无胸部放射治疗史，无化疗和生物治疗史；有可测量或可评价的病灶；外周血中性粒细胞≥1.5×10^9^/L，血小板≥100× 10^9^/L，血红蛋白≥100 g/L；血肌酐、胆红素 < 1.5倍正常上限，转氨酶 < 2倍正常上限；确诊前半年内体重减轻≤ 10%；患者本人及家属同意并签署知情同意书。

#### 1.1.2 剔除标准

既往或治疗时合并有其它恶性肿瘤（非黑色素皮肤癌或宫颈原位癌除外）；任何放、化疗禁忌的疾病或情况；恶性胸腔积液或心包积液者。

### 1.2 治疗方法

#### 1.2.1 化疗

诱导化疗采用EP方案（etoposide, 100 mg/m^2^, d1-d3; cisplatin, 80 mg/m^2^, d1）静脉点滴，3周重复；2周期化疗结束后1周-2周内行放疗。放疗中按期进行第3周期化疗。放疗后再行3周期化疗，3周重复1次。

#### 1.2.2 放射治疗

所有患者均采用增强CT定位扫描，扫描范围从第四颈椎到第二腰椎。经三维治疗计划系统设计放疗计划。靶区勾画按照ICRU62号文件的定义。研究组大体肿瘤体积-原发灶（gross tumor volume-tumor, GTV-T）按照化疗后残留的原发灶范围勾画，对照组GTV-T按照化疗前原发灶范围勾画。两组大体肿瘤体积-淋巴结（gross tumor volume-nodal, GTV-N）包括阳性淋巴结范围（CT扫描短径≥1 cm，或一个结区内有3个及以上的成簇小淋巴结，或PET/CT阳性，或纵隔镜活检病理阳性）；临床靶体积（clinical target volume, CTV）的勾画按照GTV-T外扩0.8 cm的区域为CTV-T，两组均不做纵隔选择性淋巴结预防性照射（elective nodal irradiation, ENI），CTV-N均包括治疗前达到诊断标准的淋巴结所在的结区，即化疗后完全缓解（complete response, CR）的淋巴结所在的结区仍需勾画。计划靶体积（planning target volume, PTV）为CTV外扩1 cm-1.5 cm区域。放疗总剂量45 Gy，分割剂量1.5 Gy，2次/天，间隔时间≥6 h，5天/周。放化疗结束后疗效评价为CR的患者接受全脑预防性照射（prophylactic cranial irradiation, PCI），采用模拟机定位，两侧对穿野照射30 Gy，分割剂量2 Gy，1次/天，5天/周，或25 Gy，分割剂量2.5 Gy，1次/天，5天/周。

### 1.3 随访方法

放疗中每2周（30 Gy）时复查胸部正侧位片。治疗后4周-6周，之后每3个月随诊1次，满2年后每半年随诊1次，随诊时行常规体检及胸部X光，必要时胸部、上腹部增强CT扫描。生存时间以诱导化疗开始时间计算，至死亡时间或末次随访时间2009年11月30日。

### 1.4 疗效及毒性评价

两疗程化疗后、放疗后及巩固化疗后均行胸部增强CT检查评价疗效。采用WHO实体肿瘤客观疗效评定标准，分为CR、部分缓解（partial response, PR）、疾病稳定（stable disease, SD）及疾病进展（progressive disease, PD）。放疗期间记录肺及食管急性反应、体重变化，每周复查血常规至少1次。体重减轻和血液学急性毒性按NCI CTC AE 3.0标准评价，肺、食管急性和后期毒性反应则按RTOG标准^[16]^进行评价。

### 1.5 研究设计与统计方法

本研究设计为前瞻性完全随机化对照研究，主要观察指标为局部控制率。估计研究组和对照组3年局部控制率均为80%，取双侧值α=0.05，β=0.2，采用非劣效性检验，临床界值δ=-10%，两组样本比例为1:1，两组患者分别各需198例。采用SPSS 13.0软件进行数据处理，采用*Kaplan-Meier*法分析总生存、无进展生存数据。分组因素水平间的比较采用*Log-rank*法检验生存时间分布是否相同。两样本均数的比较采用*t*检验。原发灶所处肺叶位置、是否中央型病变、T分期、N分期及是否纵隔型N3对照射野外复发的影响采用*Logistic*回归分析。以*P* < 0.05为差异有统计学意义。

## 2 结果

### 2.1 患者资料

2002年6月-2009年4月连续收治78例患者（目前入组仍在继续）。其中1例患者为肝癌治疗后第二原发小细胞肺癌而不入组。适合入组的77例患者随机分组后的临床特征见表 1。两组患者各临床特点具有可比性。

**1:** 入组患者临床特征 Eligible patient characteristics

Characteristics	Study arm (*n*=37)				Control arm (*n*=40)			*P*
	No. of Paltients		%		No. of Patients		%	
Age (years)								0.20
Median		57				56		
Range		40-75				34-75		
Sex								0.55
Male	29		78.4		34		85.0	
Female	8		21.6		6		15.0	
KPS								0.08
90	22		61.8		32		80.0	
80	15		38.2		8		20.0	
Mean FEV1(L)		2.21				2.29		0.78
Weight loss								0.25
<5%	32		86.5		38		95.0	
5%-10%	5		13.5		2		5.0	
Tumor type								0.65
Central	20		54.1		24		60.0	
Peripheral	17		45.9		16		40.0	
Stage								0.67
Ⅰ	0		0		1		2.5	
Ⅱ	2		5.4		3		7.7	
Ⅲa	11		29.7		13		33.3	
Ⅲb	24		64.9		22		56.4	
PET/CT examination	4		10.8		4		10.0	1.00

### 2.2 患者接受治疗情况及毒副反应

#### 2.2.1 化疗

所有患者均接受2周期诱导化疗。研究组和对照组分别有3例、1例患者诱导化疗后短时间内发生远处转移而接受姑息放疗和化疗。其余患者均完成1程同期化疗。研究组和对照组患者平均完成的巩固化疗程数分别为1.8±1.2和1.7±1.2（*P*=0.77）。

#### 2.2.2 放射治疗

其余患者均按计划完成放疗1.5 Gy，每日2次，共45 Gy。平均放疗总疗程时间分别为（22.9± 3.2）天（19天-31天）和（22.3±2.7）天（19天-29天）（*P*=0.30）；CTV的平均体积分别为（199.7±116.4）mL和（220.8±136.3）mL（*P*=0.48）。接受PCI的患者在研究组和对照组分别有11例和14例（*P*=0.75），而接受30 Gy/15次照射和25 Gy/10次照射的患者在两组分别为8例、3例和9例、5例（*P*=0.65）。

#### 2.2.3 放化疗急性毒副反应

放化疗期间≥3级的血液学毒性、放疗期间及放疗后急性放射性肺炎、放射性食管炎及体重减轻程度详见表 2，诱导化疗后PD的患者未纳入统计。

**2:** 两组放化疗的急性毒副反应的发生率 Incidence of acute toxic effects according to treatment arm

Toxic effect/grade	Study arm (*n*=34)			Control arm (*n*=39)		*P*
	No. of patients	%		No. of patients	%	
Haematologic toxcity≥grade 3						
Leukopenia						0.84
Ⅲ	10	29.4		10	26.3	
Ⅳ	4	11.8		3	7.9	
Thrombocytopenia						0.53
Ⅲ	6	17.6		3	7.9	
Ⅳ	5	14.7		3	7.9	
Anemia						0.89
Ⅲ	8	23.5		7	18.4	
Ⅳ	2	5.9		1	2.6	
Weight loss						0.04
Ⅰ	10	29.4		22	56.4	
Ⅱ	2	5.9		3	7.7	
Pneumonitis						0.46
Ⅰ	17	50.0		19	48.7	
Ⅱ	3	8.8		1	2.6	
Esophagitis						0.37
0-Ⅰ	23	67.6		30	76.9	
Ⅱ-Ⅲ	11	32.4		9	23.1	
Patients with PD after induction chemotherapy were not included in statistical analysis for toxic effects.							

#### 2.2.4 放疗晚期毒副反应

本研究未观察到脊髓晚期反应，放疗晚期毒副反应主要为Ⅲ度以下的放射性肺损伤及Ⅱ度以下的放射性食管损伤，前者在对照组中发生率较高，差异接近有统计学意义（表 3）。

**3:** 两组放疗晚期毒副反应 Incidence of radiation late toxic effects according to treatment arm

Toxic effect/grade	Study arm (*n*=34)			Control arm (*n*=39)		*P*
	No. of patients	%		No. of patients	%	
Pulmonary injury						0.07
0-Ⅰ	33	97.1		33	84.6	
Ⅱ-Ⅲ	1	2.9		6	15.4	
Esophageal injury						0.86
0	31	91.2		36	92.3	
Ⅰ-Ⅱ	3	8.8		3	7.7	
Patients with PD after induction chemotherapy were not included in statistical analysis for toxic effects.						

### 2.3 治疗效果

#### 2.3.1 总体治疗效果

两组患者在诱导化疗后，放疗结束后及巩固化疗后进行疗效评价，诱导化疗后PD的患者未纳入以后的疗效评价（表 4）。

**4:** 两组患者各阶段治疗后疗效 Tumor response after each stage treatment according to treatment arm

Tumor response	Study arm (*n*=37)			Control arm (*n*=40)		*P*
	No. of patients	%		No. of patients	%	
Induction chemotherapy						0.52
CR	2	5.4		5	12.5	
PR	23	62.2		24	60.0	
SD	9	24.3		10	25.0	
PD	3	8.1		1	2.5	
Thoracic radiotherapy						0.55
CR	10	29.4		9	23.1	
PR	21	61.8		24	61.5	
SD	3	8.8		4	10.3	
PD	0	0		2	5.1	
Consolidation chemotherap						0.33
CR	16	47.1		19	48.7	
PR	17	50.0		15	38.5	
SD	0	0		3	7.7	
PD	1	2.9		2	5.1	
Patients with PD after induction chemotherapy were not included in futher statistical analysis for treatment efficacy.						

#### 2.3.2 复发转移及生存情况

诱导化疗后PD患者不纳入局部复发分析，但纳入转移和生存分析。随访截止时研究组和对照组复发患者分别有11例（32.4%）和11例（28.2%）（*P*=0.80），其中单独照射野外复发分别为1例（2.9%）和1例（2.6%）（*P*=0.92），照射野外复发伴远处转移分别为2例（5.9%）和1例（2.6%），野外复发位置均为原发灶同侧锁骨上区（入组时体检和增强CT检查均为阴性而复发时经增强CT或超声检查证实）；单独照射野内复发分别为4例（11.8%）和6例（15.4%），照射野内复发伴远处转移分别为4例（11. 8%）和3例（7.7%）（*P*=0.83）。研究组有18例患者发生远处转移，其中8例为多发转移，部位分别为脑11例（61.1%）、骨4例（22.2%）、肝3例（16.7%）、肺1例（5.6%）、肾上腺1例（5.6%）、其它部位6例（33.3%）；对照组有15例患者发生远处转移，其中5例为多发转移，部位分别为脑9例（60%）、骨4例（26.7%）、肝3例（20%）、肺1例（6. 7%）、肾上腺1例（6.7%）、其它部位2例（13.3%）。

研究组和对照组患者中位生存时间分别为22.1个月（95%CI: 15.7-28.5）和26.9个月（95%CI: 16.8-37.0），1、2、3年总生存率分别为77.9%、44.4%、37.3%及75.8%、56.3%、41.7%（*P*=0.79）（图 1）；两组患者1、2、3年肿瘤无进展生存率分别为58.3%、39.6%、35.6%及67.9%、53.6%、39.7%（*P*=0.41）（图 2）。

**1:**
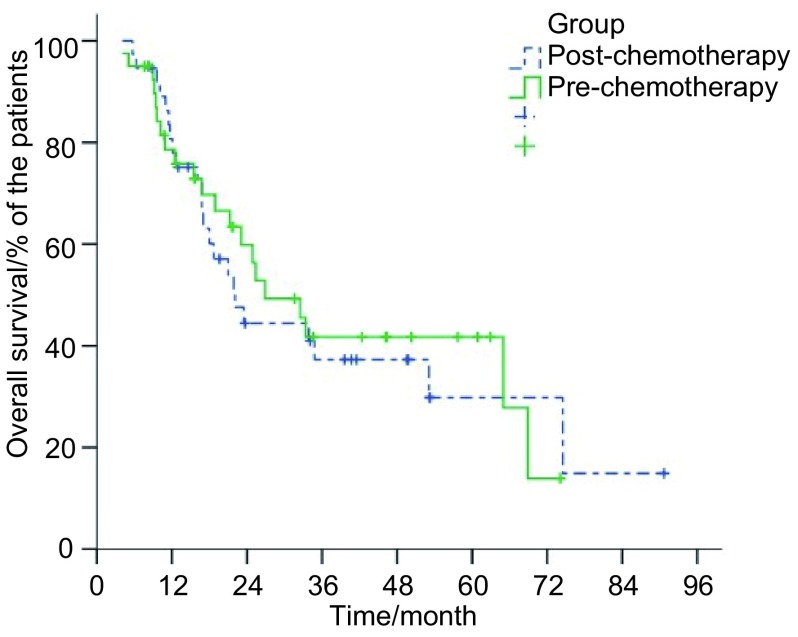
两组患者总生存率曲线 Overall survival of patients with LSCLC who were assigned to receive irradiation to the preor post-chemotherapy tumor extent

**2:**
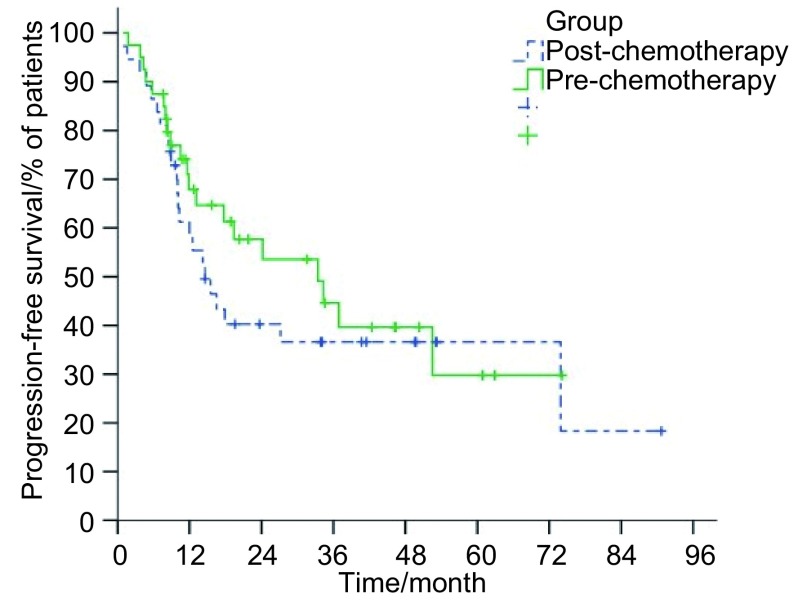
两组患者疾病无进展生存率曲线 Progression-free survival of patients with LSCLC who were assigned to receive irradiation to the preor post-chemotherapy tumor extent

### 2.4 照射野外复发相关危险因素

5例照射野外复发患者肿瘤特征见表 5。其中4例N3患者均为对侧纵隔淋巴结转移（2例为对侧第4组，1例为对侧第2组、第4组，1例为对侧第5组、第6组）。选取可能影响照射野外复发的相关因素如原发灶所处肺叶位置、是否中央型病变、T分期、N分期、是否纵隔型N3。经*Logistic*回归分析显示，纵隔型N3是照射野外复发的危险因素[*P*=0.02，优势比（odds ratio, OR）=14.13，95%CI: 1.47-136.13]。

**5:** 照射野外复发患者肿瘤特征 Tumor characteristic of out-field recurrent patients

Tumor characteristics	No. of patients (*n*=5)
Primary tumor location (lung lobe)	
Left upper	3
Left lower	0
Right upper	1
Right middle	0
Right lower	1
Primary tumor type	
Central	4
Peripheral	1
T stage	
T4	2
T2	3
N stage	
N3	4^*^
N2	1
^*^：All mediastinal N3 nodal disease.	

## 3 讨论

LSCLC放化疗综合治疗中，放疗靶区是争议的热点之一^[1, 17]^。诱导化疗后LSCLC放疗靶区的设计，又涉及到按化疗前或化疗后肿瘤范围照射以及ENI的取舍两方面。但到目前为止，仅有两项针对照射靶区的前瞻性临床研究^[18, 19]^。而二维放疗、非含铂方案化疗时代的回顾性分析研究^[20-23]^所得结论报道不一。与这两项前瞻性研究相比，本研究同时设计了随机按化疗前/后原发灶范围设置靶区和忽略ENI两方面（表 6）。

**6:** 本研究与另外两项前瞻性研究的比较 Comparison between this study and other two prospective trials

Investigators	No. of patients	Simulation method	Overall target definition
Hu *et al*	77	3D	Post- or pre-chemotherapy tumor extent, omission of ENI
Kies *et al*^[18]^	494	2D	Post- or pre-chemotherapy tumor extent, “abnormal appearing lung”，mediastinal, “low” supraclavicular fossa
De Ruysscher *et al*^[19]^	27	3D	Pre-chemotherapy tumor extent, omission of ENI

Kies等^[18]^的研究是仅有的一项Ⅲ期随机临床实验，466例LSCLC患者经诱导化疗后，疗效评价为PR或SD的191例患者中，93例、98例患者分别按照化疗前、化疗后病灶范围设野，相对应的局部复发率分别为32%和28%，差异无统计学意义，中位生存期差异也无统计学意义（51周*vs* 46周，*P*=0.73），但威胁生命的和致死性毒副反应在接受大野照射组患者中较小野照射组患者多（17/93 *vs* 8/98）。

De Ruysscher等^[19]^率先开展了对LSCLC患者忽略纵隔ENI的临床研究，结果显示11%（3/27）患者发生单独照射野外复发，超出研究者预期。但该研究样本量较小，难以从中得出明确结论。

本前瞻性研究单独照射野外复发率在研究组和对照组分别为2.9%（1/34）和2.6%（1/39）（*P*=0.92）。且照射野外复发位置均位于病灶同侧锁骨上区，与De Ruysscher等^[19]^的报告相同。但锁骨上区照射野外复发是否由于该部位复杂的解剖结构导致靶区遗漏所造成呢？研究显示PET/CT较普通增强CT能够更准确地对SCLC患者进行分期和预后判断^[24-29]^。锁骨上区淋巴结在普通增强CT上为阴性而PET/CT检出率为8.3%-12.5%^[27-29]^。但这方面仍缺乏前瞻性、大宗病例数、具备病理结果的研究。Van Overhagen等^[30]^的前瞻性研究将117例肺癌患者中锁骨上淋巴结短径≥5 mm者经B超引导下穿刺活检作为判断的金标准，比较触诊、增强CT扫描和B超在确定锁骨上区淋巴结转移的价值。结果显示增强CT（*P*=0.001）和B超（*P* < 0.001）均比触诊更能有效地诊断锁骨上淋巴结转移，而增强CT和B超相比差异无统计学意义（*P*=0.06）。

De Ruysscher等^[19]^的研究显示锁骨上复发患者均为纵隔型N3。而本研究中照射野外复发患者80%（4/5）为纵隔型N3（表 6），*Logistic*回归分析显示这类N3是照射野外复发的危险因素（*P*=0.02, OR=14.13, 95%CI: 1.47-136.13）。van Overhagen等^[30]^的前瞻性研究也发现93%（28/30）锁骨上淋巴结转移的患者在胸部增强CT上表现为N2或纵隔N3的病变，且N3患者比N0-N2患者更容易发生锁骨上淋巴结转移（*P* < 0.001），这些N3患者中发生细胞学证实的锁骨上淋巴结转移几率可达51%。

本研究中所有患者均未出现纵隔淋巴结的照射野外复发，其可能的原因在于照射肉眼可见的原发病灶和转移淋巴结的同时，照射野外的纵隔淋巴引流区接受了一定剂量的非目的性附带照射。目前已有NSCLC患者中纵隔淋巴结附带照射的剂量学研究^[31-33]^，这与纵隔淋巴结受累范围、肿瘤的位置和大小、照射野的数目和方向以及共面/非共面射野设计有关。Ronsenzweig等^[33]^的研究显示，在86%的Ⅲ期NSCLC的患者中忽略ENI，使用3D-CRT给予50.4 Gy-81 Gy照射，在上、下纵隔及隆突下区域分别有34%、63%和41%患者接受到 > 40 Gy的附带照射。但类似研究在LSCLC患者中尚未见报道。

在Turrisi等^[5]^的研究中，局部复发率在接受加速超分割放疗和接受常规分割放疗的患者中分别为36%和52%（*P*=0.06），局部复发伴远处转移率在两组患者中分别为6%和23%（*P*=0.01），而两组患者5年总生存率分别为26%和16%（*P*=0.04），这提示增加局部控制率有助于总生存率的提高。Schild等^[6]^总结了8项临床试验中所采用的有效生物剂量（biologically effective dose, BED）与长期生存的关系，*Pearson*相关系数为0.81，显示了二者较强的相关性。结合其它一些临床试验研究结果^[10, 34]^，提示我们适当增加放疗总剂量可能提高局部控制率进而延长总生存率。目前正在进行的NCT00433563临床实验^[35]^比较EP方案化疗联合45 Gy/30次/15天和66 Gy/33次/45天放疗，以及NCT00632853^[36]^比较EP方案化疗联合45 Gy/30次/15天、70 Gy/35次/47天、61.2 Gy/34次/35天放疗，其结果值得期待。

综上所述，研究结果初步显示仅照射化疗后原发灶范围及阳性淋巴结区未降低局部控制率和总生存率，而放疗毒性降低。照射野外复发均位于病灶同侧锁骨上区，而纵隔型N3是野外复发的危险因素。由于局限期小细胞肺癌占全部肺癌的比例较低，导致本研究病例积累缓慢，目前尚未达到设计要求，上述结论仍有待继续扩大样本量后进一步证实。
